# A rare case of muscular echinococcosis

**DOI:** 10.1093/omcr/omaf200

**Published:** 2025-10-29

**Authors:** P Kiskinov, A Palavurov, V Anastasova, K Atliev, E Zanzov

**Affiliations:** Department of Propaedeutics of Surgical Diseases, Section of Plastic, Reconstructive and Aesthetic Surgery and Thermal Trauma, Medical University – Plovdiv, “Saint George” University Hospital, 15A Vasil Aprilov Boulevard, Plovdiv 4002, Bulgaria; Department of Epidemiology and Disaster Medicine, Medical University – Plovdiv, “Saint George” University Hospital, 15A Vasil Aprilov Boulevard, Plovdiv 4002, Bulgaria; Department of Propaedeutics of Surgical Diseases, Section of Plastic, Reconstructive and Aesthetic Surgery and Thermal Trauma, Medical University – Plovdiv, “Saint George” University Hospital, 15A Vasil Aprilov Boulevard, Plovdiv 4002, Bulgaria; Department of Epidemiology and Disaster Medicine, Medical University – Plovdiv, “Saint George” University Hospital, 15A Vasil Aprilov Boulevard, Plovdiv 4002, Bulgaria; Department of Propaedeutics of Surgical Diseases, Section of Plastic, Reconstructive and Aesthetic Surgery and Thermal Trauma, Medical University – Plovdiv, “Saint George” University Hospital, 15A Vasil Aprilov Boulevard, Plovdiv 4002, Bulgaria

**Keywords:** muscular echinococcosis, paravertebral hydatid, parasitic infection

## Abstract

Echinococcosis is a zoonotic parasitic infection caused by Echinococcus species, with E. granulosus being the most prevalent in Bulgaria. The disease primarily affects the liver and lungs, while muscular involvement is exceedingly rare, accounting for less than 3% of cases, even in endemic regions.

We present a case of a 25-year-old male with a painful paravertebral cystic lesion three years post-surgical treatment for pulmonary echinococcosis. Imaging revealed a non-enhancing cystic mass with solid components and adjacent vertebral lysis. Due to the lesion’s location, complete en bloc excision was not feasible. A meticulous approach involving aspiration, membrane removal, and fibrous tissue excision was undertaken. The patient had an uneventful recovery and remained recurrence-free at 12 months postoperatively.

This case highlights the diagnostic and therapeutic challenges of muscular echinococcosis and underscores the importance of thorough clinical evaluation, particularly in patients from endemic regions, to ensure timely diagnosis and appropriate management.

## Introduction

Echinococcosis (from Latin: *Echinococcosis*), also known as hydatid disease or dog tapeworm infection, is a zoonotic helminthiasis caused by the larval stages of *Echinococcus* species. The disease is endemic in regions including the Mediterranean, the Middle East, South America, sub-Saharan Africa, and Australia [1]. In Bulgaria, approximately 500 new cases are registered annually.

The parasite's life cycle includes a definitive host, typically canines or other carnivores, and intermediate hosts such as sheep, cattle, or other herbivorous animals. Humans can serve as accidental intermediate hosts [2]. Adult tapeworms inhabit the intestines of definitive hosts, while larval forms (hydatid cysts) develop in various internal organs of intermediate hosts. The liver is most affected, followed by the lungs, due to the portal venous circulation through which the ingested eggs pass. Muscle involvement is rare, likely due to the unfavorable acidic environment produced by lactic acid in muscular tissue. Clinical history plays a pivotal role in diagnosis. Imaging studies, especially conventional techniques, often yield nonspecific results and can lead to misdiagnosis and inappropriate treatment [3]. Serological testing, particularly indirect hemagglutination, may significantly support the diagnostic process [4].

## Case report

**Figure 1:**
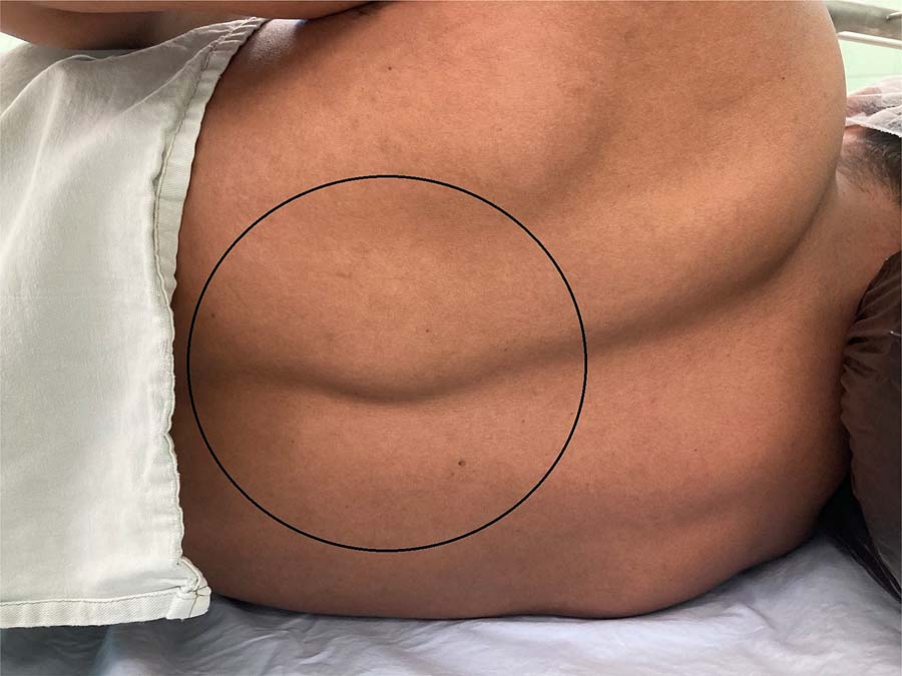
Solid formation paravertebral at the Th7–Th9 level.

**Figure 2:**
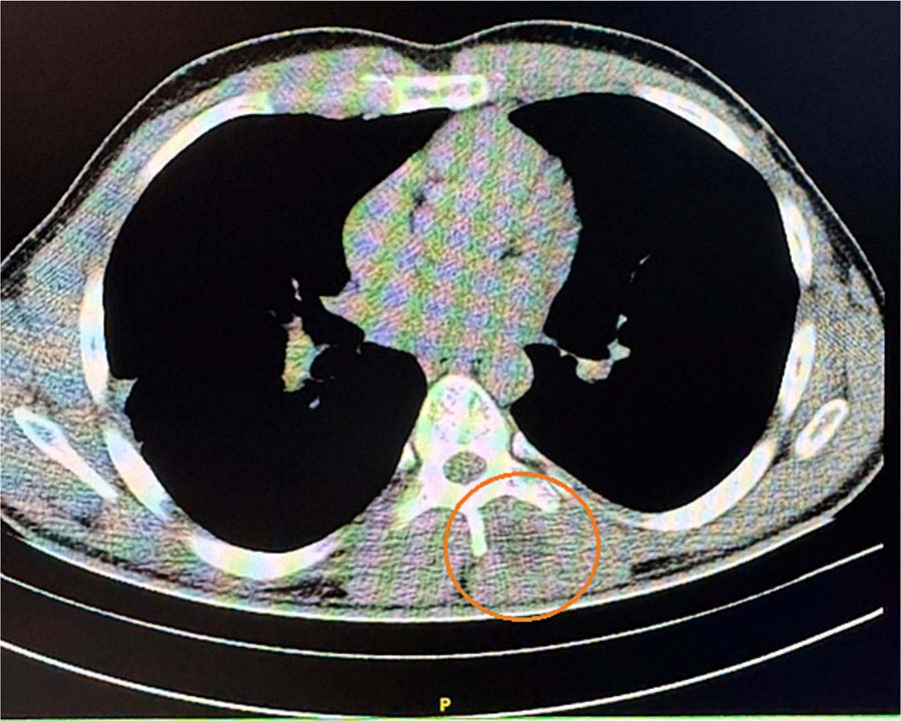
CT imaging of the formation preoperatively.

We present the case of a 25-year-old male with a painful paravertebral mass located on the left side at the level of the 8th thoracic vertebra Fig. 1. The patient reported no concurrent symptoms, no underlying illnesses, and no current medication use. Notably, he underwent surgery for pulmonary echinococcosis three years prior. There is no information on any anti-parasitical medication (e.g. albendazole) administered at that time. That could have influenced The lesion was painful upon palpation and full range of motion of the left upper limb, described by the patient as a sensation of a ``foreign body''. The mass was firm, non-fluctuant, and exhibited no signs of acute inflammation. There were no clinical or paraclinical abnormalities, and the patient denied prior trauma or pathology in the area. ELISA (Enzyme-Linked Immunosorbent Assay) was made to search for IgG: the result was negative. The complaints had persisted for several months and had intensified over the past ten days. A CT scan revealed normal lung parenchyma and a cystic lesion paravertebral at the Th7–Th9 level. The lesion did not enhance with contrast, had a density of 0–15 HU, and measured 32x32 mm. Within it, a denser area (30 HU) was identified, showing no post-contrast enhancement. Slight deformation and lysis of the articulating processes of Th8 and Th9, along with involvement of the spinous processes, were also noted Fig. 2.

**Figure 3:**
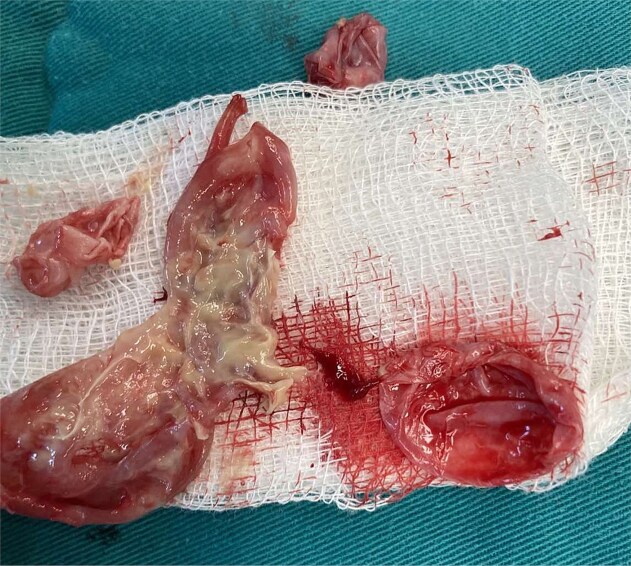
Parts of the germ and cuticular membrane.

**Figure 4:**
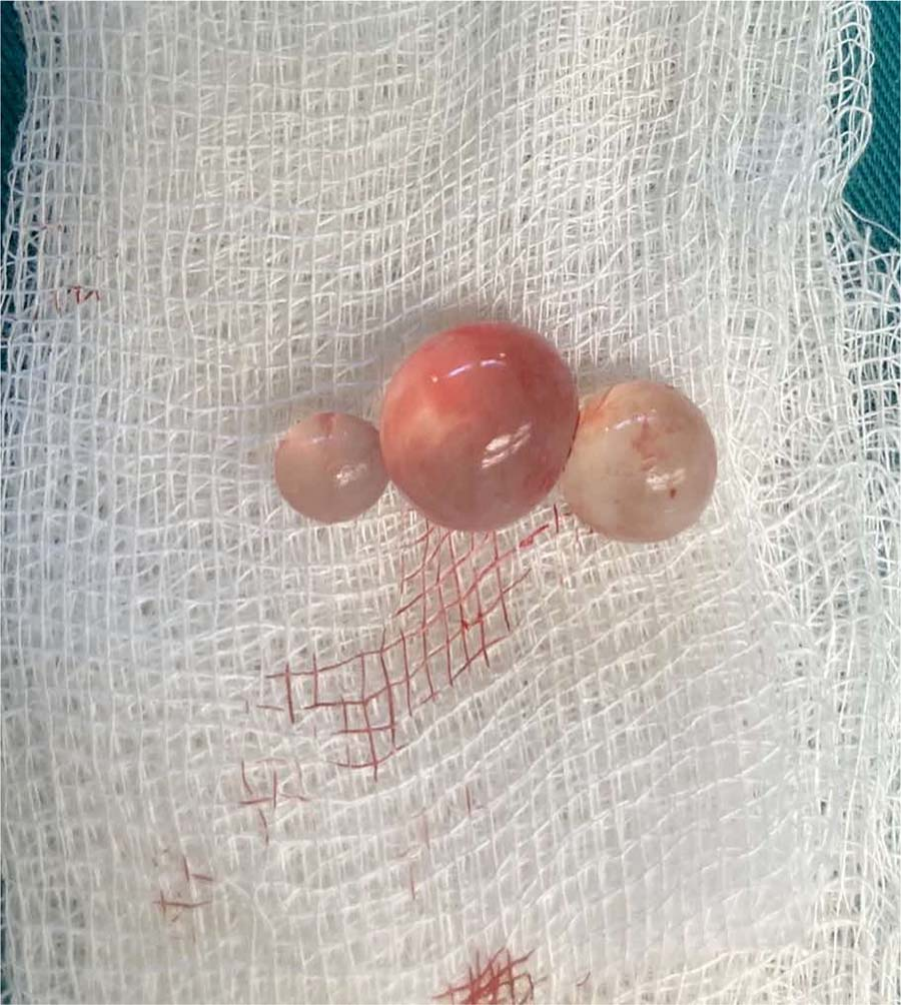
Multiple small daughter cysts.

Intraoperatively, due to the lesion's location, complete excision of the cyst and fibrous capsule without rupture was unfeasible. Aspiration and cautious exploration followed. The germinal and cuticular layers, along with the surrounding fibrous tissue and daughter cysts, were radically removed Figs 3 and 4. After meticulous hemostasis, layered closure was performed, and a corrugated drain was placed. Histopathological examination of the excised tissue was made and the diagnosis of echinococcosis was confirmed.

**Figure 5:**
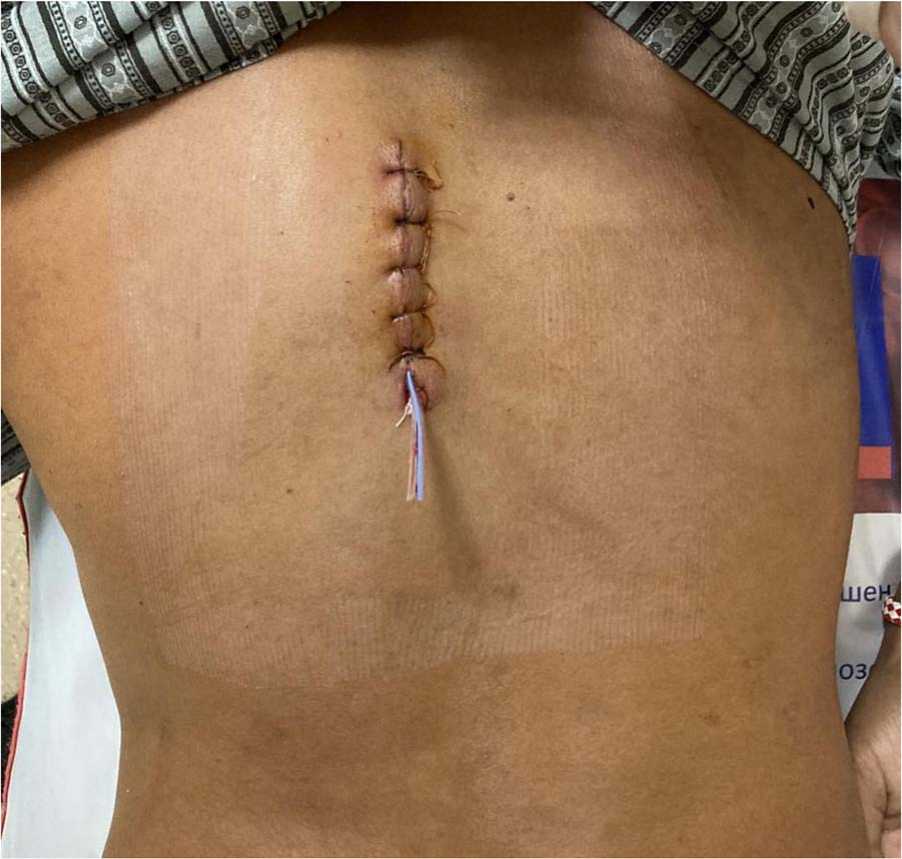
First postop. Day calm surgical wound without any secretion.

**Figure 6:**
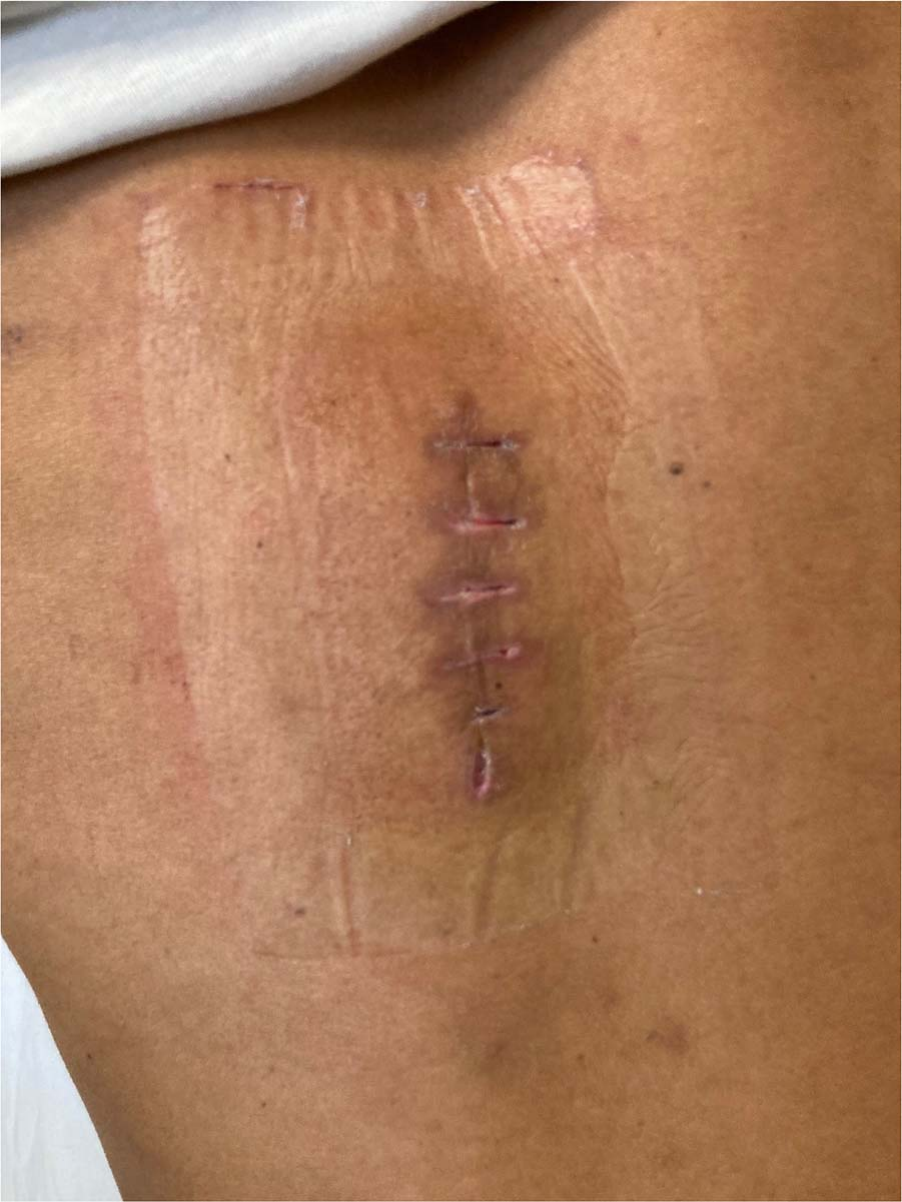
14-postoperative day. Removed sutures with good primary healing.

The postoperative course was uneventful. The wound healed by primary intention Figs 5 and 6. The patient received albendazole (Zentel) at 15 mg/kg/day for three months according to the protocol in Bulgaria as well as WHO recommendations. This will ensure sterilizing residual parasites that may have been left behind or spilled during surgery and prevents recurrence or secondary cyst development from microscopic remnants. Follow-up at six and twelve months revealed no recurrence or complications. A plan for long-term follow-up was established, but the follow-up period had not yet elapsed at the time of publishing the manuscript.

## Discussion

Of the five *Echinococcus* species identified, four can infect humans. In Bulgaria, *Echinococcus granulosus* and *Echinococcus multilocularis* are endemic, with the latter being significantly rarer and associated with alveolar echinococcosis. *E. granulosus* larvae may develop in virtually any tissue, resulting in diverse clinical manifestations [5]. Disease progression is typically slow and asymptomatic for extended periods. Some forms have a favorable prognosis when surgically treated, while others, such as osseous echinococcosis, resemble malignant bone lesions both clinically and in prognosis [6]. Vertebral and pelvic bones are most affected.

The present case involves a patient previously treated for pulmonary echinococcosis, with the initial operation conducted via an anterior-lateral thoracotomy, distant from the newly discovered lesion, thereby excluding iatrogenic dissemination. If a patient takes albendazole after surgery, the risk of recurrence or secondary cyst development from microscopic remnants is significant reduced.

We hypothesize that initial muscle involvement existed but remained undetected during earlier CT scans due to its subtle, asymptomatic nature [5]. In our case they are located beyond the area of focus in the initial scan. Muscular echinococcosis often evolves slowly, and prevalence in skeletal muscle ranges from 0.7%–0.9% [1] to 2%–3% in endemic areas [2]. It may arise spontaneously or as a delayed consequence of previous surgery. Frequently reported sites include the neck, thigh, and paravertebral region, while gluteal involvement is exceedingly rare [5]. Paraspinal muscle cases represent less than 0.5% of published echinococcosis cases [2]. One proposed mechanism for parasite translocation to paraspinal muscles involves migration through the portal circulation to the inferior vena cava, reaching the lumbar plexus during routine Valsalva maneuvers [7, 8].

Differential diagnosis includes various benign and malignant tumors, abscesses, and chronic calcified hematomas [9]. In general tuberculosis and most of the neoplasm variations show post-contrast enhancement [10]. The absence of liver or lung involvement in hydatid disease typically results in negative serological findings [11]. In our case, a detailed clinical history indicating prior echinococcosis guided both the surgical and imaging teams toward the correct diagnosis.

## Conclusion

Muscular echinococcosis is a rare clinical entity with unique therapeutic implications. Reporting such cases enriches the differential diagnosis for well-demarcated soft tissue masses. This case underscores the necessity of integrating patient history, clinical examination, serological studies, and imaging findings—particularly in endemic regions like Bulgaria—for accurate diagnosis and management.
